# The Effect of Static and Dynamic Stretching during Warm-Up on Running Economy and Perception of Effort in Recreational Endurance Runners

**DOI:** 10.3390/ijerph18168386

**Published:** 2021-08-08

**Authors:** Emanuela Faelli, Marco Panascì, Vittoria Ferrando, Ambra Bisio, Luca Filipas, Piero Ruggeri, Marco Bove

**Affiliations:** 1 Section of Human Physiology, Department of Experimental Medicine, Università degli Studi di Genova, 16132 Genoa, Italy; emanuela.faelli@unige.it (E.F.); marco.panasci87@gmail.com (M.P.); vittoriaferrando1@gmail.com (V.F.); ruggeri@unige.it (P.R.); marco.bove@unige.it (M.B.); 2 Centro Polifunzionale di Scienze Motorie, Università degli Studi di Genova, 16132 Genoa, Italy; 3 Department of Biomedical Sciences for Health, Università degli Studi di Milano, 20133 Milan, Italy; luca.filipas@unimi.it; 4 Department of Endocrinology, Nutrition and Metabolic Diseases, IRCCS MultiMedica, 20099 Milan, Italy; 5 IRCCS Ospedale Policlinico San Martino, 16132 Genoa, Italy

**Keywords:** stretching, warm-up, time to exhaustion, rate of perceived exertion, running economy

## Abstract

This randomized crossover counterbalanced study investigated, in recreational runners, the acute effects of pre-exercise stretching on physiological and metabolic responses, endurance performance, and perception of effort. Eight male endurance runners (age 36 ± 11 years) performed three running-until-exhaustion tests, preceded by three warm-ups, including the following different stretching protocols: static (SS), dynamic (DS), and no-stretching (NS). During the SS and DS sessions, the warm-up consisted of 10 min of running plus 5 min of SS or DS, respectively, while during the NS session, the warm-up consisted of 15 min of running. Physiological and metabolic responses, and endurance running performance parameters, were evaluated. The perception of effort was derived from the rating of perceived exertion (RPE). Running economy significantly improved after SS (*p* < 0.05) and DS (*p* < 0.01), and RPE values were significantly lower in SS (*p* < 0.05) and DS (*p* < 0.01), compared to NS. No differences in physiological and metabolic responses among the sessions were found. This study showed that including SS and DS within the warm-up ameliorated running economy and decreased the perception of effort during a running-until-exhaustion test, highlighting the benefits of stretching on endurance performance. These results should encourage recreational runners to insert stretching during warm-up, to optimize the running energy costs, reducing the perception of effort and making the training sessions more enjoyable.

## 1. Introduction

Runners commonly perform a warm-up before an endurance exercise, as it is considered essential to achieve optimal performance [1]. Stretching is frequently integrated into runners’ warm-up routines, but its effectiveness in improving physiological and metabolic responses, and endurance running performance parameters, is still debated [2,3].

The effects of stretching on physical performance have been demonstrated to depend on different factors, such as stretch modality [4] and duration [5], and the subject’s conditioning levels [6]. Among different stretching modalities, SS and DS are the most investigated. SS involves limb movement to the end of the range-of-motion (ROM), holding this position from 15 to 60 s [7], whilst DS requires controlled movements through the ROM, by contracting agonist muscle groups and lengthening antagonist muscle groups, without a held-end position [8]. As concerns stretch duration, recent evidence has highlighted that in SS protocols, usually designed with different durations, a clear dose-response effect exists, in which stretch durations that are longer than 60 s per muscle–tendon unit can cause performance impairments, thus adopting shorter durations is recommended. As concerns DS protocols, the current literature reported durations of up to 220 s in total as the most advisable [9].

Regarding the effects induced by SS on physical performance, some studies showed an increase in ROM around the joint, and a significant decrease in the risk of muscle injuries [10,11]. However, when investigating the relationship between stretching and injury, some studies showed contrasting data, reporting that SS was not a useful prevention strategy for endurance athletes, as it was unable to reduce the prevalence of muscular–skeletal injuries [12].

A single bout of SS during warm-up has been demonstrated to both impair running performance and running economy [3,12,13], and to reduce maximal voluntary strength and muscle power [8,14], whereas some studies reported no effect on running economy and performance following SS exercises [15,16,17,18,19,20]. Given the contrasting literature about the effects of SS on performance, an alternative modality, namely, DS, has been proposed. DS has been shown to improve power, sprint and jump performance, and enhance ROM, providing a similar, or greater, increase in flexibility than SS [8]. Furthermore, as regards the impact of DS on endurance performance, Yamaguchi et al. (2015) showed that a pre-exercise DS lasting a few minutes, acutely prolonged the time to exhaustion and extended the total running distance, without improving running economy [21], whilst other studies demonstrated no effects of DS, either on running economy [15] or on running performance [22]. In contrast, recently, the same authors showed that a combined warm-up of running plus DS impaired the immediate endurance performance, without affecting running economy [23].

Based on the current literature, the effects induced by pre-exercise stretching on endurance performance, and the most appropriate type of stretching, remain to be clarified. Therefore, in the present study, we investigated, in recreational runners, the acute effects of two pre-exercise stretching modalities on physiological and metabolic responses, endurance performance and perception of effort during an endurance running performance. To this aim, we compared submaximal continuous running and running-until-exhaustion tests, preceded by three different warm-up routines, including either static or dynamic stretching, or no stretching. Moreover, the assessment of psychophysiological stress experienced by the runners, through the rating of perceived exertion (RPE), was also a focus.

In this study, in order to clarify the discrepancies in the previous literature, we investigated whether a general warm-up plus stretching can affect physiological parameters, endurance running performance, and internal workload. Based on previous evidence, we hypothesized that the inclusion of stretching within the warm-up, followed by an appropriate resting period duration [1], would positively influence endurance running performance. In addition, it is reasonable to hypothesize that stretching exercises, given their benefits on muscle functions, could be an effective strategy to decrease the perception of effort after an endurance event. At last, considering the contrasting literature about the effects of the different modalities of stretching, we were also interested in comparing the effectiveness of static and dynamic stretching.

## 2. Materials and Methods

### 2.1. Subjects

Eight recreational male runners, not participating in systematic endurance trainings and with a weekly training volume of about 15 km/week, were enrolled.

Estimation of sample size was performed using VO_2max_ as a physiological response to exercise as one of our primary outcome measures [24,25]. Sample size was estimated using the GPower software (3.1 software Düsseldorf, Germany), applying ANOVA repeated measures (F Test) with a significant level of 0.05, a statistical power of 80% to an effect size (ES) of 0.3 [26]. This calculation generated a desired sample size of at least 6 participants. However, we recruited 8 subjects to allow for drop-out during the intervention period.

Muscle or joint injuries, orthopedic problems, severe visual impairment, cardiovascular diseases, or any other contraindication within three months before the commencement of the study were chosen as exclusion criteria. Participants’ characteristics at baseline are reported in Table 1.

Subjects were asked not to change their sport practice during the intervention period and not to exercise 48 h before the experimental sessions. They were also instructed to avoid the consumption of food in the 3 h prior to the test and to refrain from caffeine and alcohol consumption in the previous 24 h [27]. Before the experimental protocol, all subjects were fully informed about the study aims and procedure, and they gave their written informed consent to participate in the study. The study was conducted in accordance with the Declaration of Helsinki, and the protocol was approved by the Ethics Committee of Università degli Studi di Genova (protocol code: 246 and date of approval: 7 July 2020).

### 2.2. Experimental Design

A randomized crossover repeated measures design was carried out and performed at the same time of the day, one session/week.

The experimental protocol consisted of the following four sessions: a cardiopulmonary exercise test (CPET) and three endurance running sessions, differentiated by the warm-up stretching content, as follows: SS, DS, or NS [28].

Before the beginning of the experimental protocol, in order to become accostumed to the treadmill running and maximize the reliability of the running economy measurements, a “treadmill familiarization session” was completed by each participant.

During the first experimental session, subjects performed the CPET to determine their maximal oxygen uptake (VO_2max_) (mL·kg^−1^·min^−1^), used to quantify the intensity of the running-until-exhaustion session and, in a randomized order, three endurance running tests were completed (NS, SS, DS) between second to fourth session.

**Figure 1:**
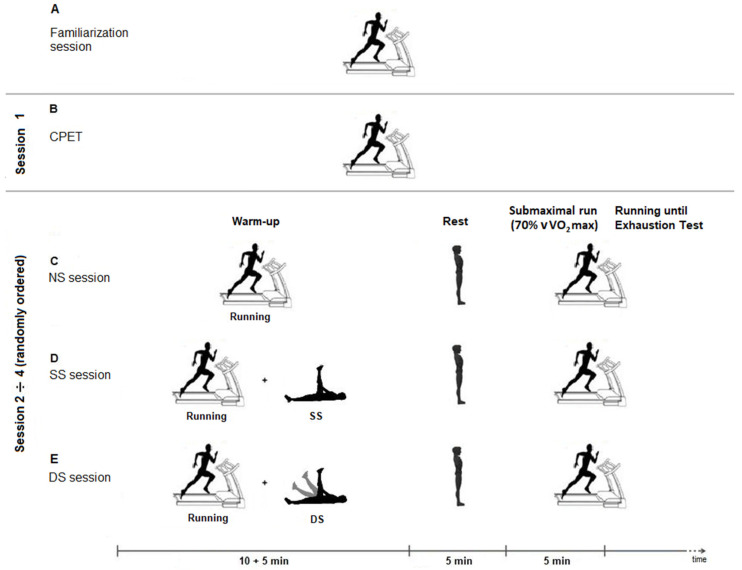
Study design. (**A**): familiarization session; (**B**): cardiopulmonary exercise test (CPET); (**C**): no-stretching session (NS); (**D**): static stretching session (SS); (**E**): dynamic stretching session (DS).

Each endurance session included 15 min of warm-up followed by 5 min of rest in a standing position [23], then 5 min of a continuous submaximal running and a subsequent running-until-exhaustion test [20]. The randomization was performed in a draw form, on the assessment day (Figure 1).

#### 2.2.1. Cardiopulmonary Exercise Test

Subjects ran 5 min at 7 km/h of speed as a warm-up, then they performed a maximal incremental exercise, with an initial speed of 8 km/h increased by 1 km/h every minute. The gradient of the motorized treadmill (E-motion, Runner, MTC climb 2000, Modena, Italy) was set at 1% to simulate the air resistance that athletes usually experience on an outdoor track [29]. The CPET was performed using an ergospirometer (Sensormedics, Viasys, Irvine, CA, USA) and a mask (Hans Rudolph, INC., Shawnee, KS, USA) with a dead space of 30 mL. Before the measurement, the ergospirometer was calibrated following the recommendation of the manufacturer and the analysis of expired gas was sampled breath by breath. Heart rate (HR) was recorded at 5 s intervals with a Polar heart rate monitor (Polar H7, Electro, Kempele, Finland). Moreover, 2 min after the end of CPET, blood lactate concentration [La]^+^ was measured [30].

Subject’s VO_2max_ was considered to be reached when at least three of the following criteria were fulfilled: (i) a steady state of VO_2_ despite increasing running velocity (change in VO_2_ ≤ 150 mL·kg^−1^·min^−1^ at VO_2max_); (ii) final respiratory exchange ratio (RER) exceeded 1.1; (iii) visible exhaustion; (iv) an HR at the end of exercise (HR_max_) within the 10 bpm of the predicted maximum [210– (0.65 × age)]; (v) a lactate concentration at the end of exercise ([La]^+^) higher than 8 mmol·L^−1^ [31]. Furthermore, we also administered the Borg’s CR-10 scale 2 min after the end of CPET. The mean (±SD) value of RPE was 9.25 ± 0.46. The minimal speed needed to elicit VO_2max_ was considered as vVO_2max_ [32]. The vVO_2max_ was considered as the speed corresponding to the last one-minute step completed during the CPET.

#### 2.2.2. Endurance Running Sessions

All endurance running sessions (SS, DS, NS) were performed on a motorized treadmill, in a laboratory with controlled temperature and humidity (21–24 °C and 44–56%, respectively) [33] and at the same time of the day (11a.m. ± 1 h) to avoid influence of the circadian rhythms. Before starting, subjects were fully familiarized with all the procedures. The experimental protocol was generally well tolerated, and subjects completed all the endurance running sessions without complication, not reporting dizziness, light-headiness or nausea symptoms. During endurance sessions, subjects performed 15 min of warm-up including running plus stretching (SS or DS) or not (NS), followed by 5 min of rest. At the end of the rest period, they were asked to perform 5 min of constant submaximal running at a speed corresponding to 70% VO_2max_ to evaluate running economy. The mean values of speed at 70% of VO_2max_ was 10 km/h. At the end of the submaximal running, the subject continued to run. Then, the speed was increased and the vVO_2max_ was reached in about 15 s. From this time point, the time to exhaustion (TTE) was measured with a manual stopwatch [20]. Mean values of the speed corresponding to VO_2max_ (vVO_2max_) was 14.45 km/h

During each running-until-exhaustion test, subjects were blind to time and distance achieved.

#### 2.2.3. No-Stretching Session (NS)

In the NS session, the warm-up consisted of 15 min running at an intensity corresponding to 60–70%VO_2max_.

#### 2.2.4. Static Stretching Session (SS)

In the SS session, the warm-up consisted of 10 min of running at 60–70%VO_2max_ plus 5 min of SS. The SS exercises focused on the following 5 lower limb muscle groups: quadriceps, hamstrings, hip flexors, hip adductors and gluteals [28]. All the static stretches were held at the “point of discomfort” (POD) for 30 s per leg.

#### 2.2.5. Dynamic Stretching Session (DS)

In the DS session, the warm-up consisted of 10 min of running at 60–70%VO_2max_ plus 5 min of DS. The DS exercises focused on the same lower limb muscle groups and in the same execution order as the SS protocol. Subjects repeated each DS exercise 30 s per leg and the maximal ROM was achieved, by ensuring a secondary pulling motion with each repetition.

**Figure 2:**
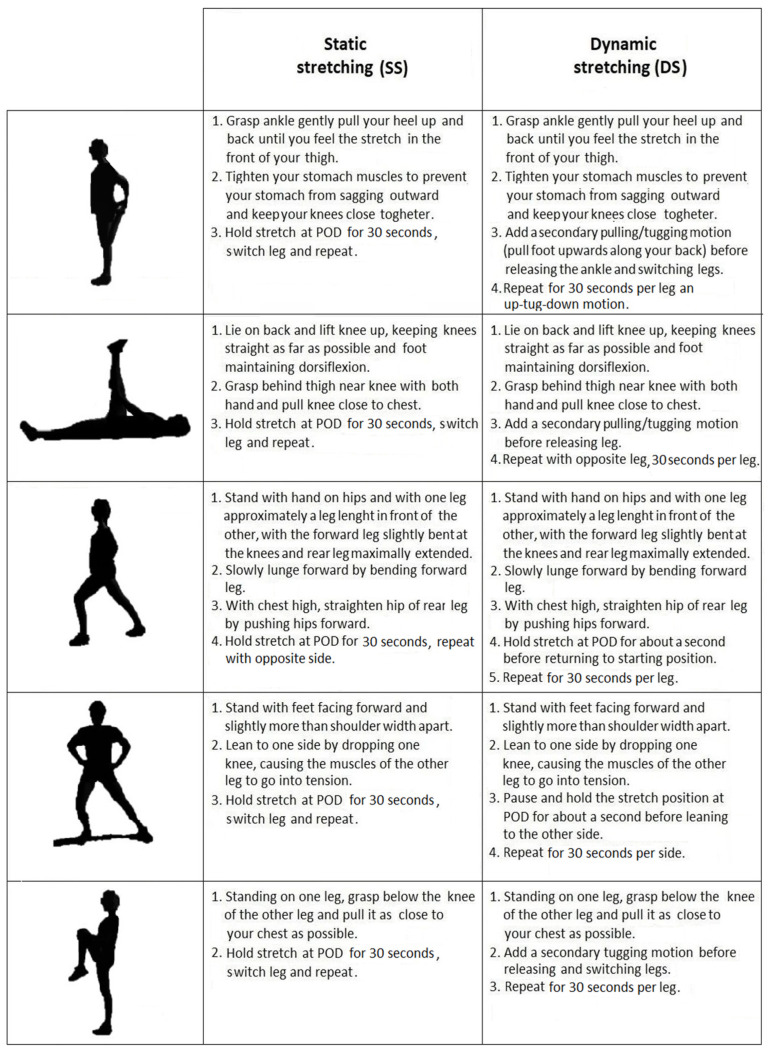
Description of static stretching (SS) and dynamic stretching (DS) exercises. Point of discomfort: POD.

Both stretch intensity (100% of the POD) and stretch volume (30 s per leg) were matched for the two stretching protocols. SS and DS exercises and instructions are shown in Figure 2.

### 2.3. Outcome Measures

#### 2.3.1. Physiological and Metabolic Parameters

Maximal oxygen uptake (VO_2max_), respiratory exchange ratio (RER), heart rate (HR), and blood lactate concentration ([La]^+^) were chosen as dependent variables to describe physiological and metabolic responses [34]. Physiological parameters were continuously monitored during the running-until-exhaustion test. The average VO_2_ value, obtained during the last 30 s of the final running stage, was considered as VO_2max_. RER was averaged over the last minute of each running velocity and HR_max_ was identified as the highest value recorded during the test [27]. Moreover, according to Assadi and Lepers [30] [La]^+^ was assessed 2 min after the end of each running-until-exhaustion test and it was measured with fingertip blood samples (5 μL), using Lactate Pro 2 (LP, Arkray KDK, Kyota, Japan). Before each collection, according to the recommendation of the manufacturer, the finger was cleansed with alcohol and allowed to air dry [35].

#### 2.3.2. Running Performance Parameters

TTE and total running distance (TRD) were assessed as indices of running performance. TTE was calculated as the subject’s continuous running time until exhaustion at vVO_2max_, while TRD was measured as subject’s total distance covered during the running-until-exhaustion test [23].

#### 2.3.3. Running Economy

Running economy (RE) was defined as the energy demand for a given velocity of submaximal running and was determined by measuring the steady-state oxygen uptake (VO_2_) and the respiratory exchange ratio (RER). RE assessment involved 5 min of continuous running at a constant speed corresponding to 70% of VO_2max_ [36]. VO_2_ and RER were averaged over the last minute of the 5-min submaximal trial. RER had to remain below 1.0 during the submaximal running for data to be included into the analysis [33].

RE was expressed as the net VO_2_ normalized per body mass per minute (mL·kg^−1^·min^−1^), where net VO_2_ is the VO_2_ measured during the submaximal running minus the resting VO_2_ averaged over the last minute of the 5-min post warm-up recovery period [36].

#### 2.3.4. Rating of Perceived Exertion

The perception of effort was assessed through the RPE, using the 11-point CR-10 scale developed by Borg [37]. The CR 10 is a category–ratio scale that ranges from 0 (no effort at all) to 10 (maximal effort ever experienced) with a dot at the end to rate an effort that is the highest that has ever been experienced. The use of RPE has been demonstrated to be related to physiological markers, such as maximal oxygen consumption and lactate, and can be used as a surrogate for heart rate to understand the heart rate response to a specific exercise intensity [38].

All the runners were informed about the RPE scale before the study started and were familiarized on the use of this scale, including anchoring procedures [39]. The validity and reliability of Borg’s scale in determining the training load of athletes, has been confirmed in previous studies [40].

Two minutes after the end of each experimental session [41], subjects had to answer the question “How intense was your session?” looking at the verbal expressions and then giving the number representing their RPE. A rating of 0 (low anchor, nothing at all) was assigned to the lowest exercise intensity, while a rating of 10 (high anchor, very, very hard) was assigned to the highest exercise intensity [38].

### 2.4. Statistical Analyses

The normal distribution of the data was checked with Shapiro–Wilk test and the sphericity with Mauchly’s test. VO_2_ rest, TTE, TRD, HR_max_, and [La]^+^ were normally distributed, whilst VO_2_ at 70% vVO_2max_, VO_2_, RE and RPE were not. Normally distributed data were analyzed by means of one-way ANOVA with SESSION (3 levels, NS, SS, DS) as factor, followed by Newman–Keuls post hoc comparisons. Effect size was described by partial η^2^ (η^2^). Non-parametric analysis, consisting of Friedman test followed by post hoc, was used to evaluate VO_2_ values at 70% vVO_2max_, VO_2_, RE and RPE values. ICC estimates and their 95% confident intervals were calculated. Effect size was described by Kendall’s test (W) [42]. The statistical analyses were performed with SPSS version 20 (SPSS, Inc., Chicago, IL, USA). Normally distributed data are means ± standard deviations, whilst not-normally distributed data are median values (interquartile interval). An alpha level of p < 0.05 was chosen.

## 3. Results

Data are offered in Table 2.

### 3.1. Physiological and Metabolic Parameters

The result of the one-way ANOVA on VO_2_ values at rest, showed that they did not differ among the conditions (F(2,14) = 0.22, *p* = 0.809, η^2^ = 0.03). The Friedman test on VO_2_ values at 70% vVO_2max_ showed a significant effect of SESSION (χ^2^(8,2) = 14.25, *p* = 0.001, W = 0.89), and post hoc comparison revealed that the DS values were significantly lower than NS (*p* = 0.001), whilst a trend in the same direction was observed between SS and NS (*p* = 0.073). The Friedman test showed that VO_2_ did not significantly change among the sessions (χ^2^(8,2) = 3.25, *p* = 0.197, W = 0.20), as well as HR (F(2,14) = 0.43, *p* = 0.66, η^2^ = 0.06), and [La]^+^ (F(2,14) = 0.48, *p* = 0.63, η^2^ = 0.06).

### 3.2. Physiological and Metabolic Parameters

The result of the one-way ANOVA showed that TTE was comparable among the three endurance running sessions (F(2,14) = 0.11, *p* = 0.90, η^2^ = 0.02), as well as TRD (F(2,14) = 0.02, *p* = 0.98, η^2^ = 0.03).

### 3.3. Running Economy

The Friedman test showed a significant effect of SESSION (χ^2^(8,2) = 13.00, *p* = 0.002, W = 0.81), indicating that RE was significantly lower both in SS (*p* = 0.037) and DS (*p* = 0.001) than in NS. Moreover, no significant differences in RE, between the SS and DS sessions, were found.

### 3.4. Rating of Perceived Exertion (RPE)

**Figure 3:**
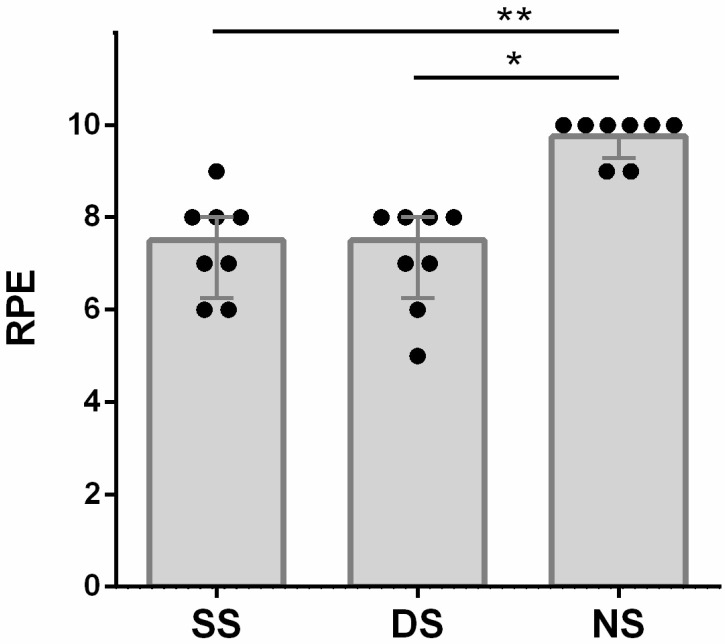
Rating of perceived exertion (RPE) measured at the end of static stretching session (SS), dynamic stretching session (DS) and no-stretching session (NS). Data are means ± SE. * *p* < 0.05 ** *p* < 0.01.

The statistical analyses revealed a significant effect of SESSION in RPE values (χ^2^(8,2) = 15.08, *p* = 0.001, W = 0.94). Post hoc tests showed that RPE in the SS (median [interquartile interval] = 7.50 [6.25, 8.00]) and DS sessions (median [interquartile interval] = 7.50 [6.25, 8.00]) was significantly lower than in NS (median [interquartile interval] = 10.00 [9.25, 10]) (SS vs. NS, *p* = 0.003; DS vs. NS, *p* = 0.018). No significant differences in RPE, between the SS and DS sessions, were found (Figure 3).

## 4. Discussion

The present study examined, in recreational runners, the acute effects of different pre-exercise stretching modalities on physiological and metabolic responses, endurance performance, and perception of effort. In particular, we compared three running-until-exhaustion tests, preceded by different warm-up protocols, including running plus stretching (static or dynamic) or running only (no stretching) routine.

The first finding of the present study was that both SS and DS exercises during warm-up, ameliorated running economy. Running economy is defined as the energy demand for a given velocity of submaximal running [25,43]. RE is complex and multifactorial, and is related to biomechanical, metabolic, neuromuscular, and cardiorespiratory factors. Improvements in RE can be achieved following interventions such as endurance training and stretching [25]. Previous literature indicated that runners with a good RE use less energy and, therefore, less oxygen uptake at the same velocity, than runners with a poor RE [43]. In the present study, recreational runners showed significant RE improvements following both SS and DS stretching exercises within the warm-up, indicating that an acute bout of pre-exercise stretching, prior to an endurance exercise, can significantly ameliorate the energy cost of running, which is probably thanks to its effects on muscle–tendon stiffness [44]. However, the current literature shows unclear results about the effects of stretching on running economy, showing a negative impact [3,11] or reporting no significant changes [15,20].

These conflicting results could be due to methodological design limitations and the different stretching durations applied in the experimental protocols.

Regarding methodological limitations, treadmill running technique is considered one of the most common in determining RE. Running on a treadmill is proven to be different from running on the ground, where wind resistance affects VO_2_ and hamstrings are used to a greater extent, to produce propulsive forces [29]. For this reason, a lack of familiarity with treadmill running mechanics could limit improvements in RE, and an adequate treadmill familiarization period is recommended for maximizing the reliability of the running economy measurement [45]. A further methodological limitation can be referred to mixed-gender experimental protocols, with minimal inferences on the underlying mechanisms, given the great differences in flexibility between males and females [45].

Additionally, different durations in stretching protocols might justify the discrepant effects of pre-exercise stretching on running economy. Recent evidence, in fact, showed that running economy improves by applying medium stretch durations (≤90 s), whereas it is not affected by long stretch durations (≥120 s) [9]. This may be due to the relative positive work of the serial elastic elements in relation to the muscle fiber work during runs at submaximal levels, thus favorably influencing RE [9].

It has to be noted that the values of RE from our study seem low when compared to other studies [25]. To explain this point, we can observe that a number of physiological and biomechanical factors, as above stated, as well as variation in the protocols, gas analysis equipment, and data averages techniques, appear to influence the assessment of RE [45]. Indeed, RE can vary among runners with similar VO_2max_ by as much as 30% [43].

A second finding of this study was that the rating of perceived exertion (RPE), measured at the end of the running-until-exhaustion test, decreased following both stretching protocols. Perception of effort is one of the most suitable representative indexes of fatigue, and can be defined as “the effort expended in performing a physical activity” [46], or as ”a conscious manifestation of the feelings of effort produced by exercise” [47]. Perception of effort refers to the discomfort that is experienced during the exercise, and is a key factor in the regulation of training intensity, particularly concerning recreational sport activities [48] and exercise to exhaustion, where high RPE values force athletes to end the exercise, although they would be metabolically and muscularly able to continue running [49]. The significant lower subjective perception of effort shown after the SS and DS sessions, suggests that a warm-up combining running and stretching exercises is more effective than a general running warm-up, so that a further endurance exercise became more tolerable and sustainable. Muscular changes occurring after stretching exercises, may better explain the effects of stretching on perception of effort. First, the altered viscoelastic properties of tendons and muscles could affect the afferent feedback to the central areas that are responsible for controlling the perception of effort and the anticipatory regulation of endurance performance [50]. Moreover, the ROM and flexibility were found to be enhanced by stretching, thanks to increased tendon elasticity and decreased muscle viscosity [51]. Also, repeated contraction and relaxation of the muscles during warm-up have been proved to enhance body and muscle temperature, increasing both the nerve conduction velocity and muscle compliance [1,8]. Recently, a cryostimulation protocol has also shown its effectiveness in improving trunk and lower limb flexibility [52], suggesting a link between the benefits on muscle flexibility and decreases in perception of effort [47].

Finally, in line with previous studies [9,12,13,20,28,53], in which longer-lasting stretching protocols were applied, our findings showed no effect either on physiological and metabolic responses or on running performance, following a few minutes of stretching exercises within the warm-up, in any of the experimental sessions tested. In particular, we showed that the time to exhaustion at the power output corresponding to VO_2max_, which is considered to be a key factor in determining running performance [15,17,32], was not significantly prolonged, and TRD was not significantly increased by pre-exercise stretching, confirming the ineffectiveness of short-duration pre-exercise stretching in improving running performance.

Our findings, however, are in contrast with two previous studies involving well-trained athletes, in which a pre-exercise DS protocol of a few minutes proved effective in improving [21], or affecting [23], running performance. In particular, the first study showed that DS exercises within the warm-up were able to increase running performance parameters during the immediate running-until-exhaustion, at an intensity close to 90% VO_2max_ [21], while the second research demonstrated that the combination of running, plus few minutes of DS exercises, as a warm-up caused running performance impairments [23]. However, the differences between our findings and those of these studies could be explained both by the different exercise intensities used in the experimental design and by the type of subjects recruited.

Based upon the absence of relevant differences between the two stretching modalities tested, we can speculate that the benefits induced by SS and DS can both effectively affect running economy and self-estimation of effort, by ameliorating mechanical, as well as energetic, properties of the muscles involved in the exercise.

Although the study involved the number of subjects required by the sample size calculation, it would be optimal to increase the sampling number.

In addition, since, under our experimental conditions, no improvements in running performance parameters during the test until exhaustion were found, it could be useful to examine these warm-up protocols on other types of endurance tests. Finally, future studies are needed to investigate whether different post warm-up rest durations would be able to improve the running performance parameters.

## 5. Conclusions

In the present study, we showed that in recreational endurance runners, both static and dynamic stretching protocols ameliorate running economy at submaximal load, and decrease the perception of effort after a running-until-exhaustion exercise.

SS is frequently used within the warm-up routine, based upon its well-known benefits, including the increase in flexibility and ROM, and the reduction in injury risk [2]. DS is reported to be an effective stretching method, on the basis of a close similarity between the stretching and exercise movement patterns, and the induced elevation in core temperature with an increase in nerve conduction velocity, muscle compliance, and, finally, in central drive [2].

We can speculate that recreational runners, by performing stretching within the warm-up, lowered running energy cost, thus making the training session more tolerable.

Finally, considering the positive effects induced by stretching prior to endurance running, the application of both SS and DS exercises, as a part of the warm-up routine, may be recommended to recreational runners. Such an approach, on one hand, may optimize the running energy cost, and, on the other hand, may reduce the perception of effort, making the training session more enjoyable.

## Figures and Tables

**Table 1:** Subjects’ anthropometric and cardiorespiratory characteristics. Data are means ± standard deviation.

Age (years)	Weight (kg)	Height (cm)	VO_2_max (mL·kg^−1^·min^−1^)
36.00 ± 11.51	71.99 ± 9.65	176.53 ± 6.36	50.13 ± 5.12

VO_2max_: maximal oxygen uptake.

**Table 2:** Physiological, metabolic and endurance performance parameters. Data are mean/median values (± standard error/interquartile interval). P values indicate the level of significance.

	SS	DS	NS	Statistics (*p* Level)
Physiological and metabolic parameters				
VO_2_ rest (mL·kg^−1^·min^−1^)	5.81 ± 0.32	5.85 ± 0.32	6.08 ± 0.26	*p* = 0.809
VO_2_ at 70%vVO_2_max (mL·kg^−1^·min^−1^)	24.70 [24.15, 25.25]	23.95 [23.05, 24.75]	29.70 [29.12, 30.58]	*p* = 0.001
VO_2_max (mL·kg^−1^·min^−1^)	50.15 [48.20, 51.58]	51.15 [50.13, 52.90]	49.70 [48.30, 51.50]	*p* = 0.20
HR (bpm)	176.63 ± 8.75	177.63 ± 10.47	176.50 ± 10.94	*p* = 0.66
[La]^+^ (mmol·L^−1^)	15.09 ± 2.43	15.18 ± 2.79	14.22 ± 3.32	*p* = 0.63
Endurance performance parameters				
TTE (s)	161.63 ± 42.16	166.63 ± 45.00	164.38 ± 36.43	*p* = 0.9
TRD (m)	666.88 ± 171.84	676.38 ± 163.16	669.88 ± 142.84	*p* = 0.98
RE (mL·kg^−1^·min^−1^)	19.05 [17.7, 20.3] *	18.20 [16.85, 19.90] **	23.40 [22.83, 24.20]	*p* = 0.002

VO_2_: oxygen uptake, HR: heart rate, [La]^+^: blood lactate concentration, TTE: time to exhaustion, TRD: total running distance, RE: running economy, SS: static stretching session, DS: dynamic stretching session, NS: no-stretching session (NS). * and ** indicate the significant differences between SS (*p* < 0.05) and DS (*p* < 0.01) with NS, respectively.

## Data Availability

The data presented in this study are available on request.
